# Molecular Epidemiology of Influenza A(H1N1)pdm09 Viruses from Pakistan in 2009–2010

**DOI:** 10.1371/journal.pone.0041866

**Published:** 2012-08-20

**Authors:** Uzma Bashir Aamir, Nazish Badar, Muhammad Rashid Mehmood, Nadia Nisar, Rana Muhammad Suleman, Shehzad Shaukat, Salman Sharif, Jaleel Kamran, Syed Sohail Zahoor Zaidi, Birjees Mazher Kazi, Larisa Gubareva, Xiyan Xu, Rebecca Garten, Alexander Klimov

**Affiliations:** 1 Department of Virology, Public Health Laboratories Division, National Institute of Health, Chak Shahzad, Islamabad, Pakistan; 2 Public Health Laboratories Division, National Institute of Health, Chak Shahzad, Islamabad, Pakistan; 3 Influenza Division, Centers for Disease Control, Atlanta, Georgia, United States of America; The University of Hong Kong, China

## Abstract

**Background:**

In early 2009, a novel influenza A(H1N1) virus that emerged in Mexico and United States rapidly disseminated worldwide. The spread of this virus caused considerable morbidity with over 18000 recorded deaths. The new virus was found to be a reassortant containing gene segments from human, avian and swine influenza viruses.

**Methods/Results:**

The first case of human infection with A(H1N1)pdm09 in Pakistan was detected on 18^th^ June 2009. Since then, 262 laboratory-confirmed cases have been detected during various outbreaks with 29 deaths (as of 31^st^ August 2010). The peak of the epidemic was observed in December with over 51% of total respiratory cases positive for influenza. Representative isolates from Pakistan viruses were sequenced and analyzed antigenically. Sequence analysis of genes coding for surface glycoproteins HA and NA showed high degree of high levels of sequence identity with corresponding genes of regional viruses circulating South East Asia. All tested viruses were sensitive to Oseltamivir in the Neuraminidase Inhibition assays.

**Conclusions:**

Influenza A(H1N1)pdm09 viruses from Pakistan form a homogenous group of viruses. Their HA genes belong to clade 7 and show antigenic profile similar to the vaccine strain A/California/07/2009. These isolates do not show any amino acid changes indicative of high pathogenicity and virulence. It is imperative to continue monitoring of these viruses for identification of potential variants of high virulence or drug resistance.

## Introduction

In April 2009, novel reassortant influenza A(H1N1) virus {A(H1N1)pdm09} emerged causing a pandemic that affected 214 countries and resulted in 18449 virologically confirmed deaths (*WHO update on Pandemic H1N1 2009, 6^th^ August 2010*). The expeditious global spread of this virus that was first detected in the United States and Mexico led the World Health Organization (WHO) to raise the pandemic alert level to phase-6 on 11 June 2009 [1].

Genetic analysis of the pandemic virus revealed a novel combination of genes from human, swine, and Eurasian avian viruses [2]–[4]. The virus carried the HA, NP and NS genes of classical swine virus origin, the PB2 and PA genes from North American avian viruses, the PB1 gene from viruses of human origin and the NA and M genes came from Eurasian swine avian-like viruses. The virus had possibly originated from triple reassortant swine viruses circulating in pigs since 1997–98 that contained human like HA, NA and PB1 genes and internal genes PB2 and PA of avian origin [3], [5]–[6]. Molecular analysis showed that these viruses had diversified into at least seven distinct clades (clades 1–7) with well defined spatial distribution [4], [6]. Furthermore, these viruses lacked specific molecular determinants of adaptation to human hosts, thereby suggesting a role of as yet unknown/undocumented molecular markers associated with human transmission [6]. These viruses did not possess markers associated with high virulence or pathogenicity that were seen in 1918 H1N1 or highly pathogenic H5N1 viruses.

The A(H1N1)pdm09 virus was found to be highly transmissible and had a distinct biological advantage in replication, transmission, tropism and pathogenesis when compared to both seasonal A(H1N1) and A(H3N2) representative viruses [6], [7]–[9]. Similarities in epidemiological behavior of this new influenza strain were observed among populations of both the northern and southern hemispheres [10]–[15]. The overwhelming majority of patients experienced mild illness with severe disease and high mortality rates in certain risk groups including diabetics, obese and pregnant women [8].

Pakistan is the world's sixth most-populous country with an estimated population of over 180 million, the second most urbanized nation in South Asia with 36% of population being now city dwellers. The first laboratory confirmed infection with A(H1N1)pdm09 influenza was detected on 18 June 2009 in a student returning from the United States. From August onwards the virus was identified in individuals who had recently returned from abroad. In early October, the spread of the virus started in the general population and the epidemic activity peaked in late December-early January in various parts of the country. With a high population density and inadequate health and diagnostic facilities, it was very important to understand the evolution of these viruses within Pakistan in comparison with viruses circulating globally. Our report presents the virological data analysis of A(H1N1)pdm09 viruses from Pakistan isolated during early epidemic period.

## Results

Between 27 April 2009 and 31^st^ August 2010, a novel influenza A(H1N1)pdm09 was detected in (262 (21%) out of 1287 suspected cases tested using Real Time RT-PCR Assay for Swine Influenza (**Table-1**). The samples negative for the pandemic strain were tested for seasonal influenza and showed a combination of influenza B (n = 180), A(H3N2) (n = 6) and non-typed Influenza A viruses (n = 49) thereby showing a total influenza prevalence rate of 38.6% (n = 497).

**Table 1:** Real-Time PCR Assay data on respiratory samples collected between April 2009 and August 2010.

Year	Samples tested	Positive for H1pdm09
**2009(April–December)**	625	152 (24%)
**2010(Jan–August)**	662	110 (17%)
**Total**	1287	262 (20%)

### Antigenic and Phylogenetic Analysis

Antigenic characterization was performed on 29 randomly selected cultured viruses in the Haemagglutination Inhibition (HI) test with use of a set of reference ferret antisera. Table 2 represents typical results of antigenic analysis of viruses collected in Pakistan during the study period. All 29 tested H1N1pdm09 viruses were characterized as A/California/7/2009-like since they demonstrated HI titers between 640–5120, thus not exceeding 4-fold difference from the average homologous HI titer (1280) of the vaccine virus A/California/7/2009.

**Table 2:** Haemmaglutination Inhibition Assay for Pakistani A(H1N1)pdm09 viruses.

	Reference Ferret Antisera		
Antigens	A/California/7/2009	A/England/195/2009	A/Brisbane/59/2007
A California/7/2009	**1280**	2560	20
A/England/195/2009	2560	**2560**	**ND**
A/Brisbane/59/2007 (seasonal H1N1)	<10	ND	**1280**
**Pakistan Isolates**			
A/Pakistan/81/2009	2560	2560	ND
A/Pakistan/154/2009	5120	5120	ND
A/Pakistan/27/2009	2560	ND	<10

ND: Not Done.

**Figure 1:**
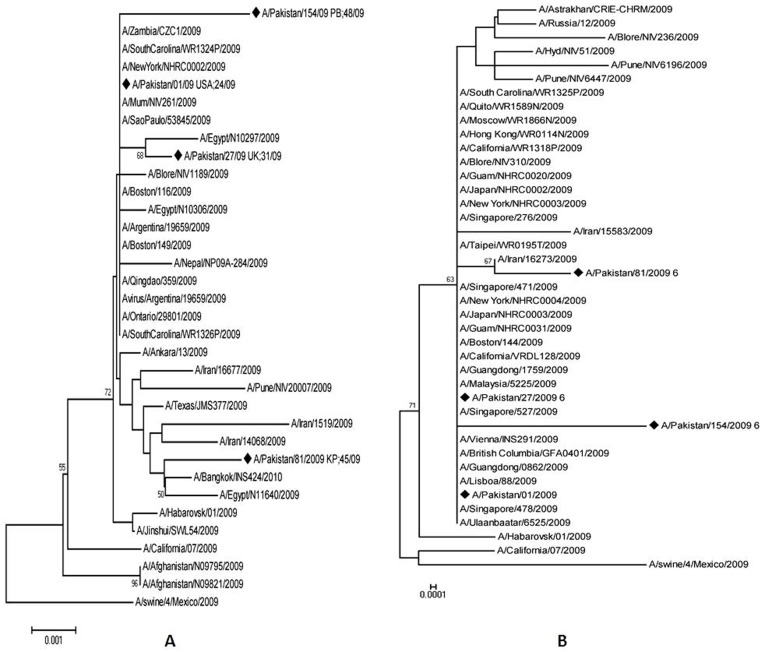
Phylogenetic Analysis for Haemagglutinin (HA) and Neuraminidase (NA) genes: Phylogenetic trees for HA and NA genes of Pakistan pandemic H1N1 2009 viruses. Full length sequences are included in the phylogenetic tree. The horizontal lines are proportional to the number of nucleotide changes. The trees were constructed using the Neighbor-Joining method using the Tamura 3-parameter model with Mega software version 4.

Four A(H1N1)pdm09 viruses detected between June–December 2009 were sequenced for surface glycoproteins and matrix genes and whole genome sequencing was carried out for two out of these four isolates; A/Pak/81/09 and A/Pak/154/09. All patients whose viruses were sequenced made a full recovery without any complications or sequelae. The nucleotide and deduced amino acid sequences were compared with A(H1N1)pdm09 sequences from various countries available in Gen Bank database. The Pakistan 2009 pandemic viruses showed >99% sequence similarity to regional 2009 novel viruses from India, Iran, China and the reference vaccine strain A/California/07/2009 (Figs. 1a and 1b). The percentage divergence from A/California/07/09 at amino acid level was 0.55% for the HA gene and only 0.35±0.12% amongst Pakistan isolates.

Analysis of Pakistan isolates for clade specific positions placed these viruses in clade 7 with characteristic mutations reported earlier for various genes [4], [6]. Substitutions in the HA (S203T) and NS1 (I123V) genes were observed in Pakistan viruses as summarized in Table 3.. Substitutions at D222G and D239G in Haemagglutinin (HA) that are reportedly associated with severe disease [10], [16], [17] were not found in the analyzed viruses. Fourteen randomly selected viruses showed histidine (H) at position 275 in Neuraminidase (NA) gene, indicating sensitivity to neuraminidase inhibitors. Other substitutions V106I and N248D in NA gene were present in the analyzed viruses (Table 3).

**Table 3:** Comparison of gene sequences of Pakistan H1N1 viruses with regional and global H1N1 isolates at key amino acid position in HA, NA, NS1 and PB1 genes.

Gene Position Isolate #	HA					NA			NS1		PB1	
	83	203	222	293	321	106	248	275	123	154	591	627
A/California/07/09	P	S	D	Q	I	V	N	Y	I	G	R	E
A/Pak/01/09	**S**	**T**	-	-	V	I	D	-	V	-	-	-
A/Pak/27/09	**S**	**T**	-	-	V	I	D	-	V	-	-	-
A/Pak/81/09	**S**	**T**	**N**	-	-	I	D	-	V	-	-	-
A/Pak/154/09	**S**	**T**	-	-	V	I	D	-	V	-	-	-
A/India/NIV6196/09	S	T	-	-	V	I	D	-	V	-	-	-
A/India/NIV310/09	S	T	-	-	V	I	D	-	V	-	-	-
A/Afghanistan/NO9795/09	S	-	-	-	V	I	D	-	V	-	-	-
A/Iran/1519/09	S	T	G	-	I	I	D	-	V	-	-	-
A/Singapore/O901/09	S	T	-	-	V	I	D	-	V	-	-	-
A/Finland/614/09	S	T	-	-	-	I	D	-	V	-	-	-
A/Finland/692/09	S	T	-	-	-	I	D	-	V	-	-	-
A/Australia/61/09	S	T	-	-	V	I	D	-	V	-	-	-
A/Malaysia/8860/09	S	T	N	-	V	I	D	-	V	-	-	-
A/Oslo/INS110/09	S	T	-	-	V	I	D	-	V	-	-	-

These viruses were also uniformly resistant to amantadine and rimantadine because of the S31N substitution in the M2 gene.

The PB2 gene had Arginine (R) at position 591, but the E627K or D701N changes known for highly pathogenic avian viruses were not observed. The D92E substitution in the NS1 gene associated with more virulent human viruses was not seen in the Pakistan isolates [17]–[19].

Phylogenetic analysis of the HA gene (Figure 1a) showed that Pakistan viruses dispersed throughout the tree and did not appear to have diverged further from other regional viruses. A similar pattern was observed on phylogenetic analysis for NA gene, and the Pakistan viruses dispersed uniformly in the tree (Figure 1b). The internal genes of Pakistani isolates also exhibited high levels of sequence identity at nucleotide and amino acid level to A(H1N1)pdm09 sequences from neighboring countries available in Gen Bank (such as India, Iran, China and Egypt).

### Glycosylation sites of HA and NA molecules

Potential glycosylation sites in the HA and NA molecules of study isolates were analyzed for changes in comparison with the vaccine strain. (Eight potential glycosylation sites were reported in the HA molecule of the A/California/07/2009 virus at positions 27, 28, 40, 104, 293, 304, 498 and 557). However, no new potential glycosylation sites were found in the either HA or NA genes of Pakistani viruses when compared with A/California/07/2009.

## Discussion

In a span of few weeks, A(H1N1)pdm09 viruses had diversified sufficiently to form 7 distinct clades, although the epidemiological behavior of these viruses was largely uniform with certain risk groups (such as pregnant women, diabetics, obesity) more prominently vulnerable than others [4]. These viruses were detected in Pakistan as early as June 2009. The earlier cases were travel importations during June–August 2009, and increased number of domestic infections was observed from September 2009 to March 2010.

Present study demonstrates that even the earliest imported isolates clearly belonged to clade 7. All the sequenced viruses belonged to clade 7 with signature change S203T, and no clade 5 or 6 that first appeared in Asia in May–September, 2009 [6], [16] were found in this study. Since the first confirmed cases from Pakistan were reported relatively late (June; week 13), and the limited number of viruses sequenced in this study; this may partly explain the presence of only clade 7 isolates in current analysis which were prominent in Asia from week 9 onwards.

The epidemiological data (not shown here) demonstrated that the early cases in Pakistan were imported through foreign travelers; however soon after that indigenous evolution and transmission resulted in widespread of the virus. The presence of a large number of mild or sub-clinical cases combined with a limited surveillance network for case detection may explain the relatively low number of reported and confirmed A(H1N1)pdm09 cases in Pakistan.

The antigenic profile of all tested 2009–2010 Pakistan isolates showed that these viruses were antigenically close to the A/California/7/2009 vaccine virus. Therefore, the advisability of identifying risk groups through serological studies and developing appropriate vaccination strategies cannot be overstressed.

Diagnostic use of real-time PCR assay has already proven to be highly effective in the detection of seasonal influenza viruses. The rapid development and prompt dissemination of the real time PCR diagnostic assay for detection of swine influenza A (H1N1) virus was instrumental in institution of rapid response and control measures across the Global Influenza Surveillance Network (GISN). The typical period during which the virus may be detected with the use of real-time RT-PCR is 6 days (whether or not fever was present at the time of sample collection). In the absence of a fully robust and proficient surveillance and sample delivery capacity, we believe that many asymptomatic or mildly symptomatic cases may have escaped testing and treatment.

The sequence data of the Pakistan viruses showed a high homology for all eight genes to the A(H1N1)pdm09 viruses from neighboring countries and to A/California/07/2009 (nucleotide identity ranged from 99–100%). The isolates group indistinguishably with other viruses on phylogenetic analysis. There is no evidence of gene reassortment between pandemic strain and co-circulating seasonal influenza H1N1 or H3N2 viruses during this time period [7]. One isolateA/154/09 had a prominent long branch on HA tree since the number of analyzed viruses is quite small. As more viruses are sequenced and added to GenBank, the apparent gaps/distances observed will become clear.

Certain amino acid substitutions such as D222G in HA have been reported in connection with severe disease and poor outcome [16], [20], [21]. As none of the Pakistan viruses analyzed in this study had this substitution or a fatal outcome, their significance merits further study especially in more serious cases reported from Pakistan. One Pakistan isolate, A/Pak/81/09 showed D222N substitution that has been reported for some H1N1 viruses from Netherlands and Malaysia (Table 3). Only one isolate, A/Pakistan/81/2009 retained isoleucine (I) at position 321 in HA, while the rest of the remaining three viruses; A/Pak/1/09, A/Pak/27/09 and A/Pak/154/09 - had I to V change at residue 321 that was seen in certain European viruses [19]. In contrast to D222G change in the HA [17], the significance of retaining isoleucine at this position for disease severity has not been clearly demonstrated. More recent substitutions such as E374K and S451N reported in isolates from Iran, Netherlands and India [16] were absent in the analyzed Pakistan viruses

In the NA gene, the clade 7 specific substitutions (V106I and N248D) were uniformly seen in the analyzed viruses while the characteristic substitutions reportedly associated with drug resistance were absent. Other substitutions such as S95N and R257K reported previously in some viruses from Finland were not present in Pakistan isolates.

Two amino acid substitutions in the PB2 gene at positions 627-K(Lysine) and 701-N (Aspargine) have been reported as important in adaptation of avian influenza viruses to mammalian hosts. Pandemic 2009 viruses that contain the PB2 gene of avian origin appear to lack these adaptive changes, however their transmission and replication efficiency is comparable to those of human viruses. Recent studies report that a basic amino acid substitution (R or K) at position 591 of PB2 counterbalances for the lack of E627K change and allows the A(H1N1)pdm09 viruses to replicate efficiently in mammalian hosts and in humans [18], [22], [23]. There are numerous as yet unexplained factors that made this novel virus highly transmissible as opposed to a highly pathogenic H5N1 that has shown poor human to human transmission capacity despite being around for over 14 years.

One of the impediments in understanding the severity of this epidemic in Pakistan is the paucity of available patient data. Out of the reported 262 positive cases, 52 hospitalized cases and 29 deaths were confirmed to be caused by Pdm H1N1 associated pneumonia and complications (Epidemiological data not shown). The viruses analyzed in the present study came from cases that recovered completely. These viruses were randomly selected for sequencing and isolates from cases with adverse or fatal outcomes are currently being subjected to detailed analysis for any significant mutations associated with high virulence. In the following months since the end of pandemic, no new reassortant events or key changes have been observed in these viruses.

Antiviral susceptibility surveillance conducted by the WHO GISN has shown that resistance for oseltamivir was sporadically detected in these viruses with rare onward transmission. None of the fourteen viruses analyzed in the present study showed the H275Y substitution associated with resistance to neuraminidase inhibitors which is not surprising since majority of cases that were virologically confirmed presented in outpatient clinic as influenza Like Illness(ILI) and most likely were not treated.(Epidemiological data on antiviral treatment is unavailable). Furthermore, data on prophylactic use of antiviral medication in suspected cases from Pakistan prior to diagnostic confirmation is currently limited, and therefore hinders analysis of potential non responders to oseltamivir therapy.

As more epidemiological and sequencing data on Pakistan influenza viruses becomes available, a better understanding of their continuing evolution will be achieved. In particular, it will be established whether any reassortment events with local seasonal influenza viruses may have occured. The continued surveillance of A(H1N1)pdm09 viruses through the coming years can ensure early detection of new antigenic or drug resistant variants. This will facilitate better pandemic planning and response capacity at national as well as global levels.

## Materials and Methods

The study was approved by Institute's Internal Review committee. A formal consent was obtained from each subject; however, the patient identities have not been disclosed at any stage. Verbal consent was taken as the study setting involved sampling of suspected pandemic cases. The institutional committee was informed of the specific needs with reference to study setting and approved this mode of consent from study subjects. A check box was included in the data form to document the consent taking procedure.

### Case definitions

The case definitions recommended by WHO guidelines were used for probable and confirmed cases [1], [3]. Briefly a probable case was defined as an individual with an acute febrile respiratory illness with disease spectrum from influenza-like illness to pneumonia and positive test for influenza A that was unsubtypable for seasonal influenza and a confirmed case with laboratory confirmed swine influenza A(H1N1) [A(H1N1)pdm09] virus infection by real-time RT-PCR and/or viral culture.

### Collection of samples and epidemiological information

During the early months of the pandemic, samples were collected from individuals with either travel history to areas of high prevalence and/or close contact with a confirmed case. Throat, nasopharyngeal swab/Broncho Alveolar Lavage (BAL) samples from suspected cases were collected in viral transport medium (Virocult®, Medical Wire & Equipment, UK). The samples were transported on ice to the National Influenza Centre NIH for Real-Time Reverse Transcriptase Polymerase Chain Reaction (rRT-PCR). Relevant epidemiological data was collected for each case on a standardized form.

### Laboratory Diagnosis (Real-time RT-PCR)

The respiratory samples were tested using Real-time RT-PCR protocol and reagents supplied by the United States Centers for Disease Control and Prevention (CDC) [24]. Briefly, RNA was extracted using QIAamp viral RNA mini kit (Qiagen, Valencia CA, USA). Real time RTPCR (rRT-PCR) for Detection and Characterization of Swine Influenza was performed on ABI -7500 using a panel of oligonucleotide primers and dual labeled hydrolysis (Taqman®) probes according to the CDC protocol. The assay tested each sample for Infleunza A (for universal detection of type A influenza viruses), Pdm Influenza A for specific detection of all swine influenza A viruses and Pdm Influenza H1 to specifically detect Pdm H1 influenza. Human RNase P gene served as an internal positive control for human nucleic acid. No template or negative controls (NTC) and positive template controls (PTC) for all primer/probe sets were included in each run. A specimen was considered presumptive positive for pdm influenza A/H1 if both the InfA and the respective subtype (Pdm InfA or Pdm H1) reaction growth curves crossed the positive threshold line within 40 cycles.

### Antigenic Characterization of A(H1N1)pdm09 Viruses

The antigenic profile of 29 A(H1N1)pdm09 randomly selected viruses was analyzed by the Haemmagglutination Inhibition (HI) assay [25] using a panel of reference post-infection ferret anti-sera raised against reference A/California/07/2009 and A/England/195/2009 viruses.

### Sequencing

Prior to sequencing, the PCR products were purified with a QIAquick PCR purification kit (QIAGEN). The purified DNA was sequenced using a BigDye Terminator cycle sequencing kit (Applied Biosystems, California) and analyzed by the ABI Prism 310 automatic sequencer (Perkin-Elmer, Foster City, Calif.). The nucleotide sequences obtained from this study are available from GenBank under accession numbers EPI355210 to EPI355228.

### Genomic sequence analysis

Sequence data were compiled and edited using the Wisconsin Sequence Analysis Software Package version 10.0 (GCG, Madison, WI). Nucleotide and deduced amino acid sequences were aligned and Phylogenetic trees constructed by using the Mega program version 4.0 [26].

### Antiviral Resistance patterns

The antiviral susceptibility testing for amantadines and neuraminidase inhibitors was carried out at CDC Influenza Division for 14 Pakistan H1N1 viruses. Amplified M gene sequences were analyzed for amino acid substitutions at the 5 positions implicated in resistance to adamanatanes (positions 26, 27, 30, 31 and 34) within the trans-membrane domain of the M2 protein [27], [28]. The presence/absence of substitutions at these positions are considered as molecular markers of resistance/susceptibility to adamantanes (amanatadine and rimantadine).

The susceptibility or resistance to neuraminidase inhibitors was assessed using the neuraminidase inhibition assay. In addition, the sequence analysis was performed presence/absence of substitutions associated with resistance to neuraminidase inhibitors, Oseltamivir and Zanamivir. (H275Y amino acid change in N1 neuraminidase corresponds to H274Y substitution in N2 numbering)
